# Safety and Quality in Maternal and Neonatal Care: Any Progress since Ignaz Semmelweis?

**DOI:** 10.3390/healthcare10101876

**Published:** 2022-09-26

**Authors:** Françoise Vendittelli

**Affiliations:** 1 Centre Hospitalier Universitaire (CHU) de Clermont-Ferrand, Centre National de la Recherche Scientifique (CNRS), Institut Pascal, Université Clermont Auvergne, F-63000 Clermont-Ferrand, France; fvendittelli@chu-clermontferrand.fr; 2 Audipog, Université Claude Bernard Lyon 1, Laennec, 7 rue Guillaume Paradin, CEDEX 08, 69372 Lyon, France

## 1. The Need to Learn from History…

James Lind (1716–1794) is considered the pioneer of medical technology assessments [1]. In 1836, Pierre Charles Alexandre Louis (1787–1872) invented the “numerical method”, which entailed following every patient’s course by scrupulously noting all their clinical variations. He thus introduced quantitative methods into the field of health. In particular, he assessed bloodletting in several diseases with the numerical method and concluded that it was ineffective (1838) [2,3]. The practice of bloodletting finally fell out of use in the 1850s. PCA Louis is considered to be the founder of evidence-based medicine (EBM).

Ignaz Philippe Semmelweis (1818–1865) worked methodically to reduce maternal deaths from puerperal fever, a bane in European maternity units in the 19th century [4]. Only after his death was he recognized as a pioneer of antisepsis and the prevention of nosocomial infections. He died in an insane asylum of blood poisoning, perhaps caused by a beating there. It was Joseph Lister (1827–1912) and Louis Pasteur (1822–1895) who finally succeeded in convincing the medical community of the usefulness of antiseptic and aseptic procedures [5]. His discoveries reduced mortality from puerperal fever, and he created the first vaccine for rabies. This revolutionized medical practice. Today, however, antivaccine lobbies have grown increasingly numerous, including in France.

Ernest Amory Codman (1869–1940) worked in Boston, Massachusetts, for the first 30 years of the 20th century [6]. He advocated making public the results of medical care, especially in hospitals. In 1900, he changed the “exit” register in place in Massachusetts hospital since the 1760s (with its categories of “cured”, “relieved”, “incurable”, “died”, “dismissed”): he was interested in the long-term outcome, the “End Result”. Thus, he added to his studies of patients at their one-year visit variables about medical errors and adverse effects: “perfect results”, “good results”, “no improvement”, “bad results”, “died”, and “died after leaving the hospital”. He is the pioneer of outcome measures that have helped to advance the quality and safety of patient care in place in several Western countries.

Archibald Leman Cochrane (1909–1988) advocated the use of randomized control trials to make medicine more effective and efficient [7,8]. He is known as one of the modern fathers of evidence-based medicine. In 1979, he wrote, “It is surely a great criticism of our profession that we have not organised a critical summary, by specialty or subspecialty, adapted periodically, of all relevant randomised controlled trials” [8]. His concept led to the establishment during the 1980s of an international collaboration to develop the Oxford Database of Perinatal Trials.

## 2. But People Do Not Always Remember History!

We are now in the 21st century: what can we say about this important work by our deceased colleagues? Beyond the fact that no one is a prophet in his own country and that they often won no glory in their lifetimes, we are compelled to observe that the effective practical implementation of their recommendations has taken time. Accordingly, lemons were not made mandatory on His Majesty’s ships until 1789 [1]. The US Congress did not create the Office for Technology Assessment (OTA) to inform it of the impact and costs of health technologies until 1972. In France, the ANDEM (national agency for the development of medical assessment) was only created in 1989. More seriously, women and children across the world still do not receive adequate care. Accordingly, there are still women with a threatened preterm delivery who do not receive antenatal corticosteroid therapy to avoid neonatal respiratory distress, although the randomized trial by Higgins et al. dates back to 1972 [9,10].

The ramping up for evidence-based medicine dates only from the second half of the 20th century. During the first half of that century, the diffusion of information was certainly not as simple as today, and that may explain in part the slowness of changes in practices in the health field. This can be illustrated by the scandal of the discovery of diethylstilbestrol (DES) at the end of the 1930s by Charles Dodds [11]; it was lauded by OW Smith [12] and very widely prescribed in the United States, where four million women were treated from the end of the 1940s through 1971. In France, 200,000 women were treated from the 1950s through 1977. Nonetheless, by 1953, a study by Dieckmann [13], a Chicago gynecologist, had cast doubt on its efficacy. In 1971, or around 20 years after the first DES prescriptions in the USA, Arthur L Herbst [14], a doctor in Boston, observed that seven young women in the region, aged from 15 to 22 years old, had been diagnosed with clear-cell vaginal adenocarcinoma and that six of their mothers had been treated during their pregnancy with DES. The story does not stop there, since the third generation is facing in turn the consequences of this drug’s use.

What can we say about the nosocomial infections throughout the world that are principally propagated by health care providers? Thus, in France, approximately one patient in 20 contracts an infection while hospitalized. It is estimated that more than 130,000 individuals—patients, health care workers, and visitors—contracted COVID-19 in a French health care facility in 2020. A procedure as simple as correctly washing one’s hands is not always adhered to, including in rich countries. Thus, some colleagues continue to struggle and seek to overcome both cultural and organizational resistance to handwashing [15].

We are in the era of genome sequencing, artificial intelligence, data mining, surgical robots, etc., and medical prowess is frequently underlined in the media. However, it was estimated in 2000 that between 44,000 and 98,000 people in the United States were dying each year through medical error [16]. Imagine how many such deaths there must be around the world. The improvement of the quality of maternal and neonatal care remains a priority worldwide, including for developed countries. Indeed, numerous serious adverse events related to perinatal and neonatal health care occur each day, during investigations, treatments, medical or surgical acts; and many result in death, the occurrence of a permanent functional impairment, prolonged hospitalizations, psychological consequences, and patient dissatisfaction. They also increase the cost of care. According to the WHO, around 300,000 women worldwide die during and following pregnancy and childbirth each year. The main direct causes of maternal death and severe morbidity are blood loss, infection, hypertension, unsafe abortion, and a long labor [17]. Most maternal deaths and severe morbidity are preventable with timely management by a well-trained health professional working in a secure environment. For the WHO, “5.1 million babies are stillborn or die in their first month of life” and “the three main causes of death are prematurity, intrapartum-related complications, and sepsis” [17]. These findings raise ethical problems. Substandard care, which can result from a lack of familiarity with evidence-based medicine and delayed recognition of the severity of the clinical context, also increases maternal and neonatal mortality and morbidity [18,19].

## 3. Then What Can We Do?

We need to ensure for all women and childs around the world safer care. This requires that health professionals are well-trained (by simulation training, etc.), have at their disposal high-quality local protocols, have clinical pathways for referrals, perform clinical audits to monitor compliance with the guidelines, improve working conditions and organizational practices, and team communication is good [20,21,22,23].

Severe maternal and neonatal morbidities should be considered as quality markers, or more precisely, indicators of missed opportunities for optimal prevention and/or the management of complications during pregnancy, childbirth, and neonatal care; they are thus important keys for preventing severe maternal or neonatal morbidity [24,25]. To think that having national guidelines is enough to improve practices is wrong [25,26]. We need good methodological and updated national guidelines and adequate local protocols adapted to the local organization to reduce substandard care [25,26]. Local opinion leaders alone, or in combination with other interventions, can be effective in promoting evidence-based practice, but the effectiveness varies between studies and the impact on patient outcomes is uncertain [27]. We also need to teach the concept of patient safety in medical universities and schools [28]. Another important issue is the organization of continued education for all health care workers.

It is also time to take patients’ experiences into account more seriously in health care systems to reduce health-care-related safety risks. Patients and families can be viewed as detectors of near-misses and other problems: just listen to them! During pregnancy at every contact with a midwife, the mother’s risk should be assessed and the management plan reviewed and updated if required. Unfortunately, this is not always well executed in many countries. The times have changed, and the role of patients must evolve from passive actors of medical care to active actor, empowered and informed co-architects of health. Nonetheless, the effectiveness of patient involvement in safety management must be assessed [29]. This is truly important because patients’ pathways are becoming more complex in the 21st century. Patients are being discharged earlier from hospitals after surgery or delivery, which is beneficial for reducing nosocomial infection and antimicrobial resistance but presents new risks, in part, because of no or insufficient coordination of care, except by the energy of the patient and/or their family, when available [30].

We now have an understanding of epidemiological prevalence and of the etiologies of adverse events, and we know the efficacy or effectiveness of some interventions. However, we need research (and therefore grants), to find interventions able to substantially improve the safety of healthcare systems [31]. More research is needed to understand the reasons for gaps in clinical practices and to identify interventions to address them [32]. We also still need more research about the best ways in which patients can change professional practices and about the impact patients’ participation has on their health [33]. Research should focus on strengthening the evidence about the effectiveness of methods to change organizational culture to improve health care performance [34,35].

The improvement of physical and psychological maternal and neonatal health all around the world must remain the major goal for medical policies and perinatal care providers. For that, safer healthcare should be developed, based on mapping the risks and benefits of any care, all along the patient’s pathway through the healthcare system [36]. We also need clinical journals to agree to publish research about safety and quality, especially, in maternal and neonatal care because perinatal practitioners do not read nonclinical journals frequently. Journals must also agree to publish studies that show the absence of any statistically significant differences between interventions. That is, the existence of a significant difference in an etiological study does not mean that there is a causal relation between exposure and the disease studied.

The main aim of this Special Issue is to aid professionals of the perinatal period and health policy makers by shedding light on the importance and the methods of improving safety and quality in maternal and neonatal care. This should be the challenge of the 21st century for perinatal care providers and for health policy makers all around the world.
